# Effect of IoT-based power cycling and quadriceps training on pain and function in patients with knee osteoarthritis: A randomized controlled trial protocol

**DOI:** 10.1097/MD.0000000000031841

**Published:** 2022-12-16

**Authors:** Xiao-yi Wang, Su-hang Xie, Yu-jia Zhang, Si-yi Zhu, Rui-shi Zhang, Lin Wang, Yuan Feng, Wei-ran Wu, Dan Xiang, Yuan Liao, Cheng-qi He

**Affiliations:** a School of Rehabilitation Sciences, West China School of Medicine, Sichuan University, Chengdu, P.R. China; b Department of Rehabilitation Medicine Center, West China Hospital, Sichuan University, Chengdu, P.R. China; c Department of Rehabilitation Medicine, First Medical Center of Chinese PLA General Hospital, Beijing, People’s Republic of China; d Department of Rehabilitation Medicine, The First People’s Hospital of Shuangliu District, Chengdu West China (Airport) Hospital, Sichuan University, Chengdu, P.R. China; e Department of Rehabilitation Medicine, People’s Hospital of Qingbaijiang District, Chengdu, P.R. China; f The Retired Office of Sichuan University, Chengdu, P.R. China.

**Keywords:** exercise, knee osteoarthritis, power cycling, quadriceps training, tele-rehabilitation

## Abstract

Knee osteoarthritis (KOA) is a chronic musculoskeletal disease affecting the entire joint. Exercise therapy is the core treatment plan for non-surgical treatment of KOA, and tele-rehabilitation is also applied to KOA, but there is a lack of research on the comparison of pain and function recovery between different exercise methods combined Internet respectively. The study aims to compare the effects of power cycling and quadriceps training combined with online guidance separately on KOA mitigation of pain, recovery of function, quality of life, and adherence of participants in the community, compared to the control group.

**Methods:**

This study is a single-blind, 12-week parallel randomized controlled trial. Seventy-two participants aged ≥ 50 years with KOA will be randomized into either the power cycling group, the quadriceps group or the control group. The intervention will be performed three times per week during 12 weeks. Outcome measures will be assessed at baseline, and at 4, 8, and 12 weeks after allocation. The primary outcome will be self-reported pain, assessed with the Western Ontario and McMaster Universities Osteoarthritis Index (WOMAC) pain subscale. Secondary outcomes will include mitigation of knee pain, quality of life, improvement of functional physical performance, adherence of participants.

**Discussion:**

By summarizing the study’s strengths and limitations, this trial results may guide tele-rehabilitation of KOA in the community.

Trial registration: The study was registered in the clinical trial registry ChiCTR2200059255, 27/04/2022.

## 1. Introduction

Knee osteoarthritis (KOA) is a chronic disease involving the whole joint, including cartilage degeneration, bone remodeling, osteophyte formation, and synovial inflammation, which could lead to knee pain, stiffness, deformity and mobility impairment, reducing patients’ quality of life.^[1]^ A recent meta-analysis showed that the overall pooled estimate of symptomatic KOA prevalence in China is 14.6%.^[2]^ The prevalence of KOA grows in the aging and obese population in China.^[3]^ The disease not only imposes a significant disease burden on individuals, but also brings a serious economic burden to the national healthcare system.^[3,4]^

Exercise therapy, as core therapy for the non-surgical treatment of osteoarthritis, has been recommended by various guidelines.^[5–7]^ As a common form of aerobic exercise, power cycling uses the structure of existing fitness bikes, with which participants can get trained by overcoming frictional resistance, and achieving the purpose of exercise.^[8]^ There are some mechanisms of power cycling that relieves joint pain, such as remodeling of brain structure and function, compression of articular cartilage and reduction of lower limb weight-bearing.^[9–15]^ Therefore, it is often recommended for community-based rehabilitation of patients with KOA.^[16]^ However, its impact on quality of life is unclear, and the optimal exercise regimen is yet to be determined. Quadriceps training is a series of simple non-weight bearing open chain exercises to improve muscle strength and endurance (especially the main knee extensors), reduce stress on the knee joint, and improve the stability of the knee, thereby attenuating joint pain and improving function in patients with KOA.^[17,18]^ One study found that quadriceps training could alleviate pain in patients with KOA, but the training effect depends on the content of exercise training, the frequency and duration of intervention.^[19]^ There is currently no research to show which training strategy is best. Furthermore, at-home training, complex exercises could reduce the compliance of participants.^[20]^ Therefore, it is particularly important to choose quadriceps training that is reliable and easy to learn. Recent clinical trials have shown that both exercise regimens could significantly alleviate pain and improve joint function^[12,21,22]^; however, whether there is a significant difference in pain relief and function improvement between the two exercise types remains controversial.^[23]^

Despite knowing the benefits of rehabilitation and exercise therapy, it is not fully utilized in reality due to the demand of the time, geography and saturation of healthcare resources_._^[24]^ To break the geographical and time constraints of rehabilitation, tele-rehabilitation emerged in 1998,^[25]^ which can conduct health education, online assessment, exercise training and follow-up.^[26,27]^ With the development of modern technology, the platform of tele-rehabilitation has gradually transitioned from the telephone to the Internet and the emerging Internet of Things (IoT).^[28]^ IoT is a network based on and extending the Internet, with which the doctors can contact the patients and make adjustments to the therapy strategies individually.^[29]^ At the same time, IoT devices can collect patient data and store it in the cloud, enabling faster and more accurate data for sharing and analysis.^[30]^ A previous study conducted by our research team summarized the first IoT-based Chinese model for KOA rehabilitation in China, which verified the importance of IoT in rehabilitation.^[31]^ Supalak et al^[32]^ applied IoT technology to the treatment tracking of patients with KOA, through which the system could improve the accuracy of remote measurements, increase the follow-up rate after treatment and improve patients’ quality of life. However, the exercise therapy of KOA was not addressed in this study.

Although Internet-based rehabilitation is safe and feasible in clinical applications to relieve pain in patients with KOA,^[33–36]^ there are limited differences in support for the effects of these two types of exercise when combined with online exercise.

## 2. Methods

### 2.1. Aim

The aim of this randomized controlled trial is to directly compare the effects of two different exercise modalities, power cycling and quadriceps training combined with online guidance separately on KOA mitigation of pain in patients with KOA in a community, compared to the control group. We hypothesize that there will be a difference between these two rehabilitation strategies. Our secondary aim is to compare the effectiveness of two different exercise modalities on other outcomes, including quality of life, improvement of functional physical performance, adherence of participants.

### 2.2. Trial design

**Figure 1.**
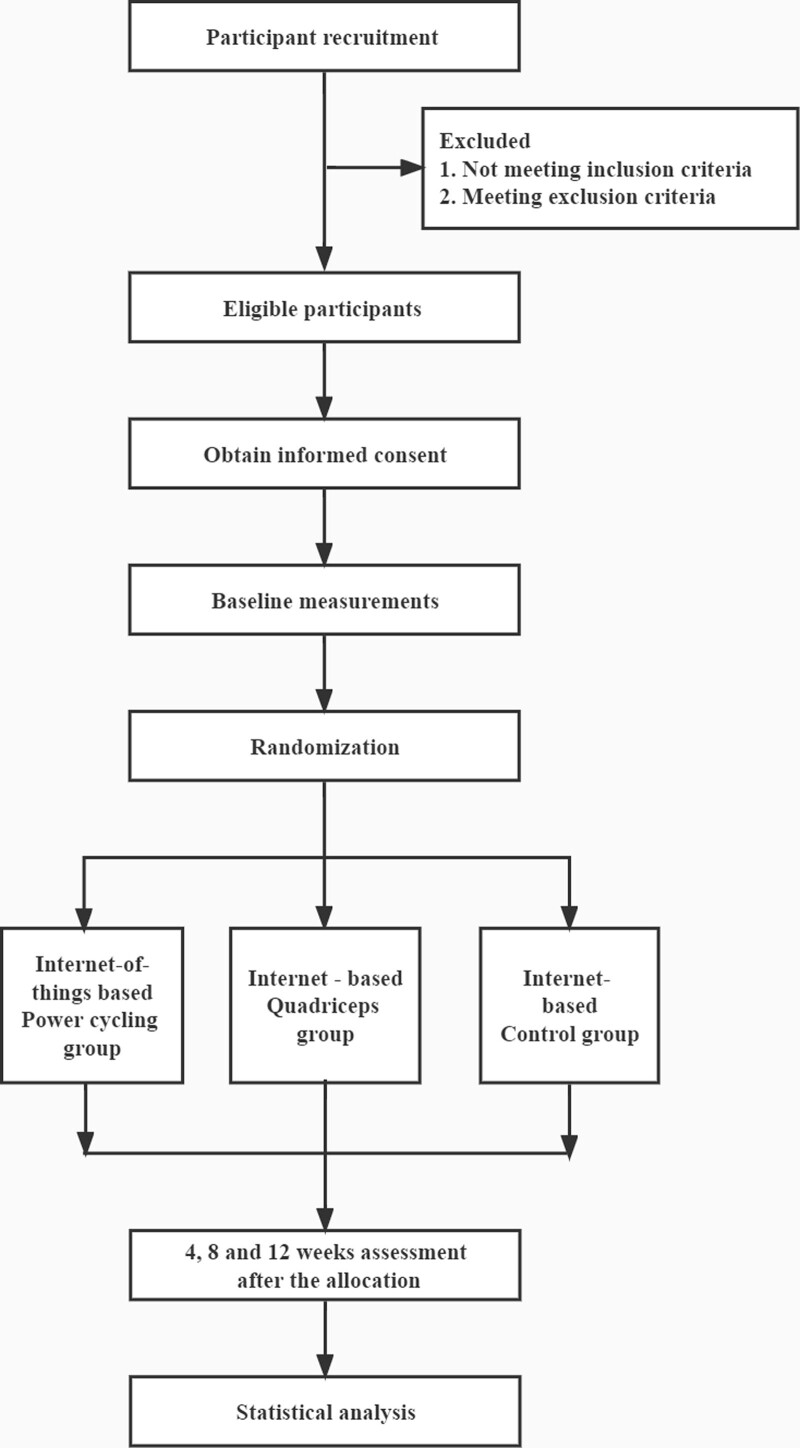
Flow chart of the trial.

This trial is a three-armed, single-blinded and longitudinal 12-week parallel randomized controlled trial. The study protocol follows the Standard Protocol Items: Recommendations for Interventional Trials.^[37]^ Meanwhile, the report will be presented as the CONSORT guidelines.^[38]^ The flow chart of the clinical trial is shown in Figure 1.

### 2.3. Study setting

This trial will be conducted in the community in Chengdu, Sichuan Province from December 2021 to December 2022.

### 2.4. Participants

We will recruit 72 patients with KOA. Participants’ inclusion criteria are: age ≥ 50 years with KOA, confirmed radiographically (Kellgren–Lawrence grade I–III), with 2021 Chinese Orthopedics Association guidelines for diagnosis and treatment of KOA clinical criteria^[39,40]^; knee pain (previous week) score of 3 to 6 on a numerical rating scale (NRS); able to use a smartphone to send messages and video calls; and able to communicate in Mandarin and understand Chinese characters.

Exclusion criteria are: having rheumatoid arthritis, gout or severe osteoporosis; preparing for knee replacement, other knee surgery in the next 6 months or knee surgery in the past 6 months; history of falls or fractures within the past 6 months; cancer; dementia or severe cognitive impairment; mental illness; diagnosis or suspicion of other conditions that limit exercise (e.g., stroke, heart failure, severe anemia, etc.); hypertension and diabetes not regularly medicated and monitored; other health problems that prevented them from participating in the project; and unable to sign informed consent.

### 2.5. Interventions

#### 2.5.1. Power cycling group.

**Figure 2.**
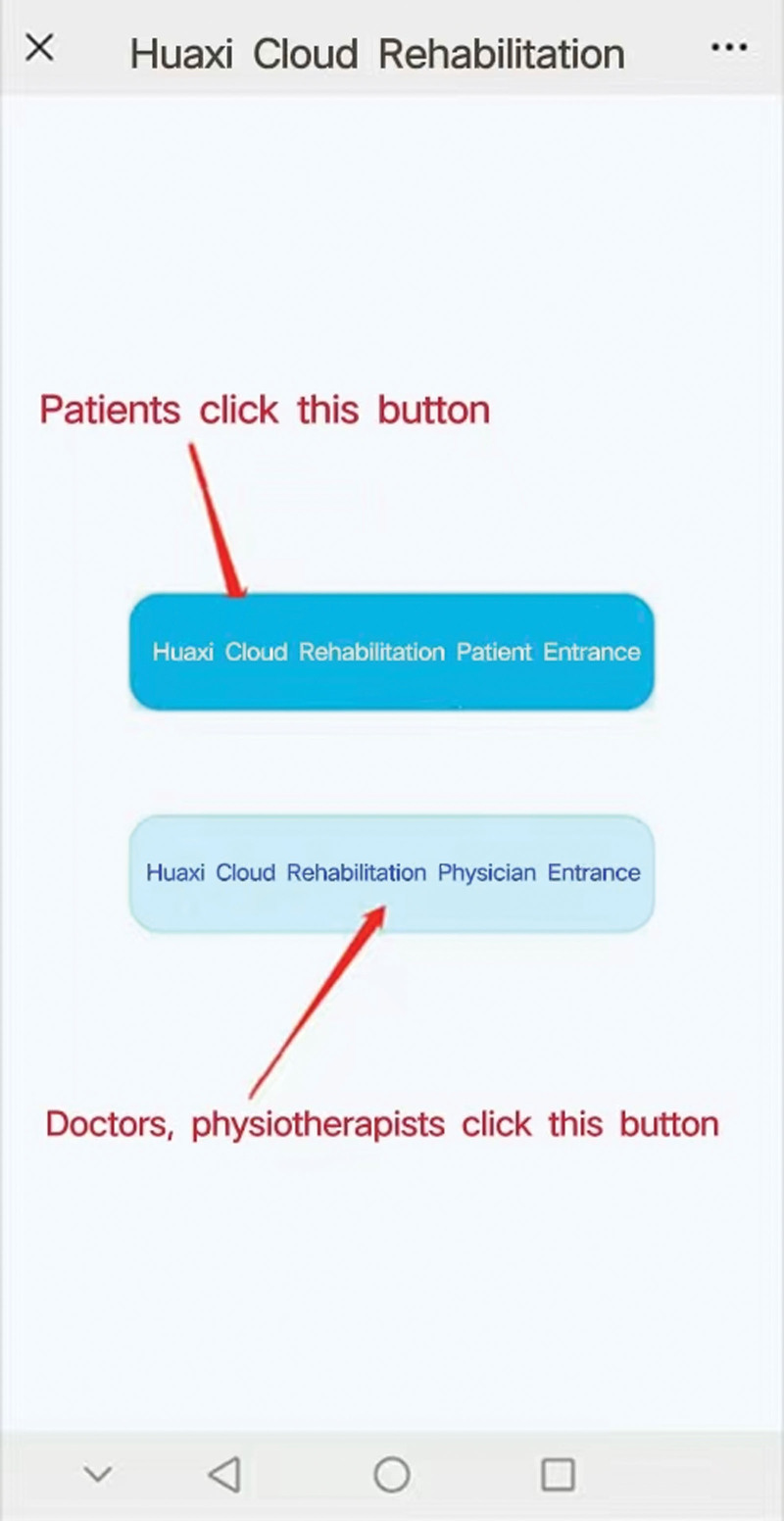
The WeChat applet “Huaxi Cloud Rehabilitation”.

**Figure 3.**
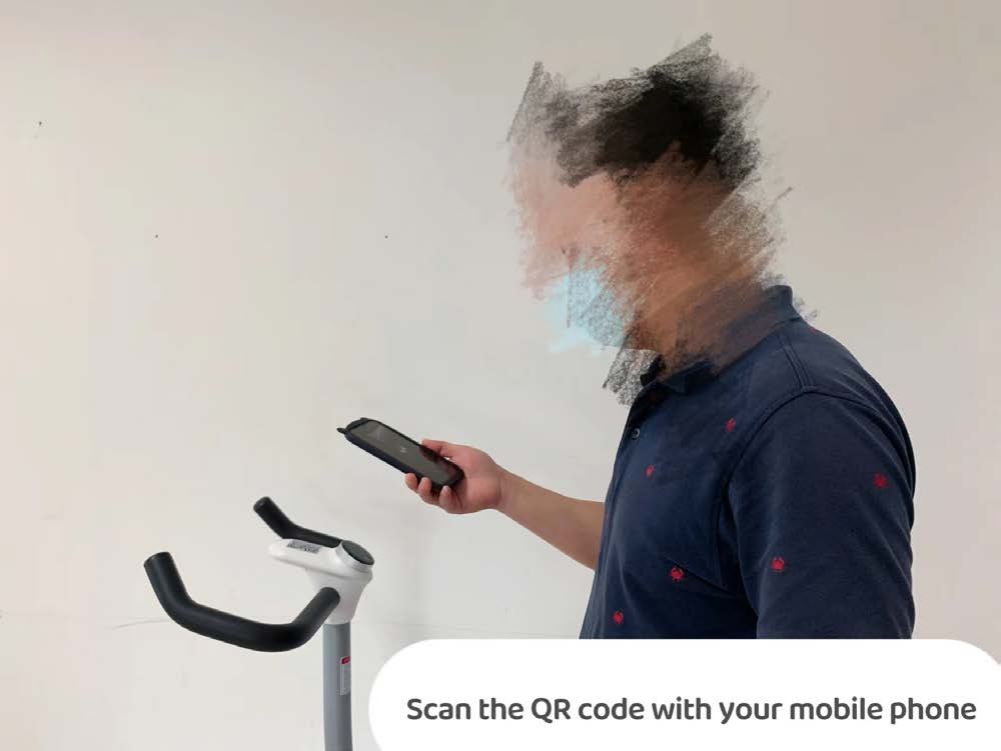
Instruction video for power cycling.

The purposely designed WeChat applet “Huaxi Cloud Rehabilitation (HXCR)” enables the conduct of IoT-based rehabilitation programs, which mainly include education, and exercise programs according to the guidelines by Osteoarthritis Research Society International.^[7]^ The WeChat applet “Huaxi Cloud Rehabilitation” for Figure 2; The power cycling for Figure 3. During the trial, glucosamine hydrochloride is also required.^[40]^

##### 2.5.1.1. Education.

By pushing knowledge about osteoarthritis disease and rehabilitation, participants will be able to understand their disease and the current status of their rehabilitation, build their confidence in rehabilitation and encourage them to actively participate in rehabilitation treatment. The doctors and physiotherapists will push educational materials related to KOA on the HXCR, including an introduction to the disease, rehabilitation treatment, healthy lifestyle (weight management, dietary management, etc.), etc. The same educational material is pushed to the participants 3 times a week, and the physiotherapist sends a relevant questionnaire to the participants at the end of each week to obtain the information received by the participants; the content is sent differently every week for a total of 12 sessions.

##### 2.5.1.2. Power cycling.

Power cycling will be performed 3 times a week for approximately 45 minutes for 12 weeks, and the exercise intensity was moderate. The intensity of exercise was measured by the rating of perceived exertion. When the rating of perceived exertion reaches 12 to 14(feeling some exertion during exercise), exercise is moderate.^[41]^ To avoid sports injuries, warm-up and cool-down activities are necessary; the time to reach exercise intensity is 30 minutes. The HXCR will send messages to the participant 3 times a week. If the participant does not complete the exercise on the same day, the message will continue to be sent to the participant on the next day, and if not completed on the next day, a message will be sent to alert the physiotherapist who will contact the participant by phone or video to check the status. Before the exercise, the physiotherapist will assess the participant and then push the exercise prescription to the participant’s applet based on the assessment results. Participants simply turn on the HXCR and scan the QR code on the power cycle to start exercising, no manual adjustments are required, and instant data (such as real-time speed, energy consumption, etc.) is displayed on the participant’s phone. A family member is required to accompany the participant during the exercise to ensure the safety of the participant. Each week, the physiotherapist will have a video link with the participant to see how he/she is doing and to record his/her response to symptoms. Exercise takes place at the community activity center power cycling parking.

### 2.6. Quadriceps group

Education and medication will be the same as the power cycling group.

#### 2.6.1. Quadriceps training.

Participants are required to perform progressive resistance strength training for 30 to 45 minutes 3 times per week for a total of 36 sessions. Physiotherapists pushed training videos to participants through the HXCR platform, and researchers guided participants through WeChat videos to ensure the quality of training. Participants need to be accompanied by their family members during each exercise session to ensure their safety. Each week, the physiotherapist will have a video link with the participant to see how he/she is doing, record his/her symptom response and decide on the amount of resistance to increase based on pain and fatigue; compliance will also be tested. According to the previous literature, we determined that there are 5 items of quadriceps muscle strength training: fixed range knee extension, sitting knee extension, sitting knee extension 30° maintenance, straight leg raise and sitting elastic band training.^[42]^

### 2.7. Control group

The control group will receive the same education and medication as the other two groups.

### 2.8. Outcome measures

Outcomes for the trial will be chosen based on Osteoarthritis Research Society International recommendations.^[43]^ Table 1 summarizes the primary and secondary outcome measures. Outcomes will be measured at baseline (T_0_) and at 4 (T_4_), 8 (T_8_) and 12 (T_12_) weeks after the allocation (Table 2).

**Table 1:** Outcomes of this study.

Measurements	Metric of measurement	Method of measurement	Time point of primary interest
Primary outcomes			
Self-reported pain	0–20 (higher scores indicate more serious pain)	Western Ontario and McMaster Universities osteoarthritis index (WOMAC) – pain subscale	baseline, and 4, 8, and 12 wk after allocation
Secondary outcomes			
Self-reported pain	0–10 (higher scores indicate more serious pain)	Numerical pain rating scale (NRS)	baseline, and 4, 8, and 12 wk after allocation
Quality of life	Standardized scores for every section (higher scores indicate more better social participation)	The MOS item short from health survey (SF-36)	
Self-reported function and stiffness	0–76 (higher scores indicate more serious dysfunction)	WOMAC – physical function and stiffness subscale	
Functional performance	Seconds (shorter time indicates better function)	Timed up and go test	
Adherence	0–100% (Higher ratios indicate better adherence)	The ratio of the number of completed HXCR sessions to the total number of sessions	12 wk after allocation

HXCR = Huaxi Cloud Rehabilitation, NRS = numerical pain rating scale, SF-36 = The MOS item short from health survey, WOMAC = Western Ontario and McMaster Universities Osteoarthritis index.

**Table 2:** The participant timeline.

Time point	Study period				
	Enrolment	Allocation	Post-allocation		
	−T_1_	0	T_4_	T_8_	T_12_
Enrolment					
Eligibility screening	×				
Informed consent	×				
Allocation		×			
Interventions					
Education					
Medication					
Exercise					
Assessment					
Sociodemographic data	×				
Personal and medical history	×				
IPAQ Short	×				
Primary outcome			×	×	×
Secondary outcomes			×	×	×

IPAQ Short = International Physical Activity Questionnaire.

#### 2.8.1. Primary outcome.

The pain subscale of the Western Ontario and McMaster Universities Osteoarthritis Index (WOMAC) will be used to assess knee pain over the past 48 hours. The WOMAC pain subscale is a patient’s self-assessed pain level consisting of 5 questions, ranging from 0 (asymptomatic) to 4 (very severe), with a total score ranging from 0 to 20, with higher scores indicating more severe pain.^[44,45]^ The intraclass correlation coefficient of the scale was 0.90, and the validity was 0.77.^[46,47]^ A previous study showed a minimum clinically important difference of 12% improvement from baseline.^[48]^

#### 2.8.2. Secondary outcomes.

Secondary outcomes are as follows:

1. NRS will be used to evaluate knee pain in the previous month. The NRS is a standard tool for the study of chronic pain. It is assessed on an 11-number scale from 0 (no pain) to 10 (severe pain),^[49]^ with 1.8 units having been reported as an minimum clinically important difference.^[50]^
2. Health-related quality of life will be assessed with the MOS item short form health survey. There are 36 questions in total, and corresponding scores are obtained according to standardized weights. The scores are added to obtain the rough test score, and the final score is obtained by the conversion formula of the scoring operation specified by the scale. The total score ranges from 0 to 100, with higher final scores indicating higher quality of life.^[51,52]^
3. Physical function assessment: The WOMAC physical function and stiffness subscale is used, with a total of 19 questions, and the score ranges from 0 to 76 points. The higher the score, the more severe the dysfunction.^[44,45]^
4. Functional physical performance includes the timed up and go test (measured in seconds, shorter times indicate better function).^[53,54]^
5. Adherence is measured at 12 weeks after the allocation by the ratio of the number of completed HXCR sessions to the total number of sessions.

Baseline assessments will be conducted after informed consent has been confirmed. The collected content included participant age (in years and based on age on ID card), gender, height, weight, BMI, education level, occupation, income level, hobbies and interests, marriage, smoking and drinking status, medical history, and level of physical activity (assessed by the International Physical Activity Questionnaire (IPAQ Short).^[55]^ During the course of the trial, if the participant’s symptoms worsen, they may be allowed to use the relevant drug for treatment. However, any medication use will be recorded in the medical record and diary.

### 2.9. Sample size

The calculation is carried out according to the standard for the sample size of noninferiority trials and the unified standard for clinical trial reporting.^[38,56]^ Based on the degree of reduction in WOMAC pain scores was used as an index to assess the improvement in symptoms of KOA with each type of exercise. The literature was reviewed to obtain the means and standard deviations of changes in pain scores in the three groups of patients with KOA,^[42,57]^ The sample content estimate formula shows that, for a significance level of 0.05 and a yielding power of 90%,


$$\begin{array}{l} n=\varphi^{2}\left[\sum_{i=1}^{k}S_{i}^{2}/k\;\right]/\left[\sum_{i=1}^{k}{\left({\bar{X}}_{i}-\bar{X}\right)}^{2}/\left(k-1\right)\;\right]\; \end{array}$$

the sample size consists of 20 participants in each group. With a 20% dropout rate taken into account, each group needs 24 enrolled participants.

### 2.10. Recruitment

Recruitment information will be made available online through various channels, such as WeChat and public numbers, as well as postings at local medical facilities, community activity centers, and the West China Hospital of Sichuan University. Two rehabilitation physicians (SX, YZ) will conduct the participant screening.

### 2.11. Randomization and blinding

After baseline measurement, participants will be randomized 1:1:1 to the power cycling group, quadriceps group or control group using simple randomization. A table of random numbers will be produced using the computer soft SPSS (V21.0). Write the random number assigned to each group on a piece of paper and place it in an opaque envelope. Based on the order in which participants were enrolled, an independent biostatistician will randomly distribute envelopes to participants. Physicians and physiotherapists will receive standardized training prior to the start of the study and will be randomly assigned to three groups during the trial. Participants won’t be blinded because of the nature of the intervention; instead, they will only know their own grouping. Evaluators, statisticians, and data managers will be blinded during the trial.

### 2.11. Withdraw and adverse events

Participants in each group may drop out of the trial. Researchers need to record the information of those who withdraw from the trial, including the reasons for withdrawal, time and other information. At the same time, the three physicians (SZ, RZ, and LW) in our department will discuss and determine the adverse events that occurred during the trial, record them on the case report form, and deal with them accordingly as needed.

### 2.12. Data management and monitoring

All outcome data will be collected by four evaluators (YF, WW, DX, and YL) at the participant’s home or community and will be stored electronically on a computer. Our department has established an electronic database to manage clinical data.^[58]^ Any accidental or adverse events will also be reported and recorded. Trial evaluators will perform data entry, and data validation will be performed by a researcher who did not perform data collection and entry.

### 2.13. Ethics and dissemination

The trial will be conducted in accordance with the Declaration of Helsinki. The trial protocol was approved by the Biomedical Ethics Committee of West China Hospital, Sichuan University (No: 2021-1642). The study was registered in the Chinese Registry website (registered in ChiCTR.org with the identifier ChiCTR2200059255). Before the start of the trial, the researchers will explain the trial protocol to the participants, inform them of the benefits and risks of the trial, and obtain the participants’ written informed consent. Participants can withdraw from the trial at any time during the trial. The results of this trial will be shared with each participant and will be presented at national or international conferences, and papers will be submitted to peer-reviewed journals.

### 2.14. Statistical analysis

Analyses will use an intention-to-treat analysis strategy. We will use one-way ANOVA to determine baseline comparability between groups for quantitative variable data. Baseline comparability between groups of categorical variable data will be determined using the chi-square test or other suitable nonparametric tests. Quantitative data will be expressed as the mean; if it follows normal distribution and homogeneity of variance, otherwise median is given. In statistical inference, if the data obeyed a normal distribution, and homogeneity of variance and satisfied spherical symmetry, repeated measures data analysis of variance was used; if the data did not obey a normal distribution or the variance was unequal, the Kruskal–Wallis H rank sum test was used for data analysis. SPSS (V21.0) will be used for data analyses. We considered *P* ≤ .05 to be statistically significant.

## 3. Discussion

KOA is a chronic disease, and its rehabilitation is also a long-term process. Tele-rehabilitation allows patients to carry out rehabilitation training in a community environment, reducing the number of patient’s visits to the doctor, saving patients’ time and money, and reducing the burden on the medical system. If effective, this study will provide important data for current tele-rehabilitation practice for KOA in community settings. The obtained data are expected to inform the development of future large-scale research trials.

Different from the previous study, the innovation of this study is the first use of IoT technology for tele-rehabilitation in KOA. Second, the multichannel recruitment of participants, especially the online publication of recruitment information, the online clinic and the online collection of basic information of participants, as well as the subsequent whole rehabilitation process (including rehabilitation assessment, rehabilitation treatment and communication with the participants), are all done online, giving full play to the advantages of the Internet and the remote nature, breaking the time and geographical restrictions and bringing the distance between doctors, therapists and patients closer.

However, some challenges also exist in the proposed study. An important challenge in this study is for participants to learn how to use IoT-based applications smoothly, although we will go into detail on first use. Second, one of the issues that need attention in tele-rehabilitation is the compliance of participants, which will reduce the compliance of the participants due to various reasons such as pain and lack of motivation of the participants. In order to increase the enthusiasm of the participants to exercise, we will remind the participants to exercise 3 times a week through a WeChat applet, send rehabilitation education materials to the participants every week, and conduct a WeChat video with the participants.

## Author contributions

CH and XW designed the trial. XW, SX, and CH drafted the manuscript. YZ, SZ, RZ, LW, YF, WW, DX, and YL contributed to the study protocol, including intervention program design, outcome measures collection and data analysis. The author(s) read and approved the final manuscript.

**Data curation:** Yuan Feng, Wei-ran Wu.

**Methodology:** Xiao-yi Wang, Cheng-qi He.

**Project administration:** Yu-jia Zhang, Si-yi Zhu.

**Software:** Dan Xiang, Yuan Liao.

**Supervision:** Rui-shi Zhang, Lin Wang.

**Writing – original draft:** Xiao-yi Wang.

**Writing – review & editing:** Su-hang Xie, Cheng-qi He.
